# Pectobacterium atrosepticum SCRI1043 flagella mediate adherence to potato plants indirectly through motility

**DOI:** 10.1099/mic.0.001588

**Published:** 2025-07-30

**Authors:** Ashleigh Holmes, Sonia Humphris, Jacqueline Marshall, Yannick Rossez, Ian Toth, Nicola J. Holden

**Affiliations:** 1 Cell and Molecular Sciences, James Hutton Institute, Dundee, DD2 5DA, Scotland, UK; 2 Université de Lille, CNRS, UMR 8576 – UGSF – Unité de Glycobiologie Structurale et Fonctionnelle, Lille, France; 3 Department of Rural Land Use, SRUC, Aberdeen, AB21 9YA, UK

**Keywords:** flagellin protein, membrane lipids, plant–microbe interactions, potato, TLC

## Abstract

Flagella are widely distributed appendages in bacteria with well-characterized functions in motility and chemotaxis. They also interact directly with hosts and, due to their ubiquity, are potent immune elicitors for hosts from both the plant and animal kingdoms. Furthermore, flagella have been shown to facilitate attachment for several different bacterial species, including several plant-associated bacteria to plant hosts. We previously demonstrated binding of flagella from *Escherichia coli* to ionic lipids in plant plasma membranes for horticultural species and *Arabidopsis thaliana*. As such, flagella could be considered as a generic colonization factor, especially in the early stages of the interactions. Therefore, we tested whether flagella from a genetically related species of plant pathogen, *Pectobacterium atrosepticum*, mediated binding to its susceptible plant host, potato, in a similar manner to *E. coli*. Surprisingly, flagella containing the filament flagellin from *P. atrosepticum* did not confer any binding advantage to potato roots. Furthermore, there was no direct interaction between purified flagella and potato membrane lipids (charged or uncharged). The binding capacity of *Pectobacterium* to potato is dependent upon the motility function of flagella, as both flagella-deficient and motor-deficient mutants were reduced in their binding to potato roots.

## Introduction

Flagella have been recognized to act as multi-functional organelles, capable of mediating binding to host tissue in addition to well-characterized roles in motility and chemotaxis [1]. Adherence to host tissue has been best described for bacterial species that interact with animal hosts, but less well for those that interact with plant tissue. Flagella have been shown to act as an adhesin for various plant-associated bacteria, including plant growth-promoting rhizobacteria *Pseudomonas fluorescens*, to the roots of potato cuttings [2] and *Azospirillum brasilense* to wheat roots via the polar flagellum [3]. They have also been shown to promote colonization of *Pseudomonas syringae* on bean seedling leaves [4]. Flagella-mediated adherence to horticultural plant species has also been described for human pathogenic bacteria: *Salmonella enterica* serovar Senftenberg [5], *Salmonella enterica* serovar Typhimurium [6], *Escherichia coli* O157:H7 [7] and *Listeria monocytogenes* [8]. In previous work, we defined the mechanism of flagella-dependent adherence of *E. coli* to plant tissue and found that it was mediated by ionic interactions between the flagellum filament and charged lipids in the plasma membrane [9]. The nature of binding, shown for three different *E. coli* flagella serotypes and on leaves and roots of three different plant species (*Arabidopsis thaliana*, *Spinacia oleracea* and *Solanum lycopersicum*), pointed to a generic rather than specific interaction. As such, we hypothesized that the interaction would extend beyond *E. coli* to other bacterial and plant host species. Therefore, we investigated whether the same mechanism of interaction occurs for a related member of the *Enterobacterales,* the phytopathogen of potato, *Pectobacterium atrosepticum* SCRI1043 (Pba1043) on potato hosts.

## Methods

### Bacterial strains, plasmids and primers

Bacterial strains, plasmids and primers are detailed in Table 1. An in‐frame *fliC* deletion mutant was constructed using pKNG101 suicide vector in *E. coli* strain CC118λpir [10,11]. Briefly, upstream and downstream regions (558 bp) of the *fliC* gene were amplified and inserted into pBluescript+before being subcloned via XhoI and ApaI digest into pKNG101 to create plasmid pAH013. The markerless exchange plasmid was introduced to Pba 1043 by conjugation as described in [11].

**Table 1.** Bacterial strains, primers and plasmids

Bacterial strain	Description	Reference
*P. atrosepticum* SCRI1043	Pba1043	[12]
Pba1043Δ*fliC*	SCRI1043 in-frame markerless deletion of *fliC* CDS	This study
Pba1043::Tn5*motAB*	Tn5 insertion 444 bp in ECA1698 (*motB*)	This study
Pba1043Δ*cheA*	SCRI1043 deletion mutant of *cheA*; Kan^R^	[36]
*E. coli* JT1	*E. coli* C600 *hsm hsr fliC*::Tn*10 fimA*::*cat*	[37]
*E. coli* DH5α	*supE44*, Δl*acU169*, φ80*lacZ*ΔM15, *hsdR17*, *recA1, endA1, gyrA96, thi-1 relA1, luxS*	Gibco BRL, Life Technologies
*E. coli* CC118λpir	*araD*, Δ(*ara-leu*), Δ*lacX74*, *phoA20*, *galE, galK, thi-1, rpsE, rpoB, argE, recA1, λpir*	[38]
*E. coli* HH26 (pNJ5000)	Mobilizing strain for conjugal transfer, TcR	[39]
**Primer**	**Sequence (5′ → 3′**)	**Purpose**
Pba.FliC.F	TCATAAGCCAGACCTCCGGGACTGG	Cloning FliC_Pba_ into pWSK29 for heterologous expression in *E. coli*
Pba.FliC.R	GCTCTAAATAAAAGAATTCGAACAT	Cloning FliC_Pba_ into pWSK29 for heterologous expression in *E. coli*
Pba_fliC_1F_XbaI	AAATCTAGAGCCTGAGCTAAATTATTCAC	Amplify the 558 bp 5′ sequence of FliC_Pba_ for KO generation Amplify across the FliC_Pba_ sequence for in trans complement in pQE80 (pJM73)
Pba_fliC_1R_SalI	AAAGTCGACGATAGCGTTCCTTAATCAGT	Amplify the 558 bp 5′ sequence of FliC_Pba_ for KO generation
Pba_fliC_2F_XhoI	AAACTCGAGTTTTCAGCCAGACAAGCGCT	Amplify the 558 bp 3′ sequence of FliC_Pba_ for KO generation
Pba_fliC_2R_ApaI	AAAGGGCCCAACACCATGAACAATAATAT	Amplify the 558 bp 3′ sequence of FliC_Pba_ for KO generation Amplify across the FliC_Pba_ sequence for in trans complement in pQE80 (pJM73)
mTn5F	ACTGTCTCTTGATCAGATCTGG	Screening the Tn5 mutant library in SCRI1043
mTn5R	TATCCTCCTTAGCTAGTCAGG	Screening the Tn5 mutant library in SCRI1043
ECA1687.mF	TGTGACGGCGGTGATAGATT	Screening the Tn5 mutant library in SCRI1043 for *motAB* mutant
ECA1687.mR	CCAGTTGCATTGGCGTAGAA	Screening the Tn5 mutant library in SCRI1043 for the *motAB* mutant
Pba_motAB_ApaI.F	ATGGGCCCGTGCAGCGGCGTTGAAGC	Amplify across the *motAB* for in trans complement in pQE80 pJM74)
Pba_motAB_BamHI.R	TTGCTCGGTATCGGGCTCCG	Amplify across the *motAB* for in trans complement in pQE80 pJM74)
pQE80.F =	CGGATAACAATTTCACACAG	Sequencing primers for pQE80 across the MCS
pQE80.R =	GGTCATTACTGGATCTATC	Sequencing primers for pQE80 across the MCS
**Plasmid**	**Description**	**Reference**
pBluescript-II KS+	High copy cloning vector, MCS in lacZ′, ApR	Stratagene
pKNG101	Suicide vector, SmR, sacBR, mobRK2, oriR6K	[10]
pAH013	558 bp upstream and downstream sequence of *fliC*_Pba_ cloned into pKNG101	This study
pQE80 (pQE80L)	Medium-copy expression vector, IPTG-inducible T5 promoter, AmpR	Qiagen
pWSK29	lac operon, low copy number (< 10) AmpR	[40]
pWSK-*fliC*_Pba_	SacI EcoRI f*liC*_Pba_ PCR cloned into pWSK29	This study
pWSK_*fliC*_H7_	*fliC* from TUV93-0 cloned into pWSK29	[9]
pJM73	pQE80_FliC_Pba_:*fliC*_Pba_ PCR cloned via EcoRI SacI digest into pQE80	This study
pJM74	pQE80_motAB:*motAB* PCR cloned via ApaI BamHI digest into pQE80	This study
pBBR	pBBR1MCS5, broad-host-range vector; GentR	[41]

The Tn5 library [12] was screened with primers mTn5F, mTn5R, ECA1687.mF and ECA1687.mR. The purified PCR products were sequenced in both directions to confirm the location of the transposon in *motAB*. The mutation was transduced into Pba1043 before analysis [13], and Sanger sequencing showed the Tn5 inserted 444 bp into the *motB* CDS. The plasmid background pWSK29 could not be used to complement the Pba 1043 mutation as it has a T7 promoter, and Pba-driven expression was more consistent via a T5 promoter for IPTG induction; hence, complement plasmids were constructed in pQE80. This has been indicated in a previous study [14].

### Heterologous FliC_Pba_ expression and antibody generation

Pba isolate SCRI1043 (Pba1043) [15] *fliC* was cloned into single-copy plasmid pWSK29 to generate pWSK-*fliC*_Pba_ and transformed into an *E. coli fliC* mutant background, strain JT1, to generate heterologous flagella. Genes involved in the regulation, synthesis and structure of flagella are well conserved in the enteric bacteria and sequence comparisons show high levels of conservation for Pba1043 and *E. coli* K-12 [16]. Flagella containing FliC_Pba_ were purified as described previously [9] from Pba1043, and 50 mg of purified flagella was used to generate polyclonal antibodies from immunization of rats (Genosphere Biotechnologies, France). Specificity of antibodies was confirmed by running flagella preparations from Pba, *Dickeya* and *E. coli* isolates on a 12.5% SDS-PAGE gel. The proteins were electrotransferred to Hybond-P membrane (GE Healthcare, Chalfont St Giles, UK), and antibody detection was carried out with anti-rat horseradish peroxidase (Sigma-Aldrich, USA). Heterologous expression of FliC_Pba_ by flagellin and type I fimbriae deficient *E. coli* strain JT1 was tested on motility agar (lysogeny broth with 0.3% agar) at 27 °C for 96 h to demonstrate restoration of motility function.

### Plant growth conditions and TLC

Potato (*Solanum tuberosum*) var Estima microplants (Gentech Propagation, UK) were cut at the internode and transplanted onto Murashige and Skoog+20% (w/v) sucrose (MS20) agar and incubated for 14 days to establish new microplants. Microplants were transplanted into 175 ml pots (Greiner, UK) containing autoclaved perlite and defined phosphate-deplete media to redistribute the relative proportions of charged and uncharged lipids as described previously [9,17]. Seedlings were grown in a cabinet with a light intensity of 150 µmol m2s-2 (16 h photoperiod) for a further 14 days at ~20 °C before bacterial inoculation or lipid analysis.

Potato roots and foliage were aseptically separated at the crown and tissue flash-frozen in liquid nitrogen for lipid analysis. Lipid extraction and 2D thin layer chromatography (TLC) were carried out on a pool of two tissue samples (Mylnefield Lipid Analysis service), as previously described [18]. Blackcurrant leaf extract was used as a standard for the localization of lipid species on the 2D TLC plate and run concurrently with the potato samples. Separated lipids on 2D TLC were sprayed with 0.01% Primulin and viewed under UV light. Potato lipids extracted from leaves (potato leaves+FliC_Pba_) or roots (potato roots+FliC_Pba_) were resolved by 2D-TLC, probed with 20 µg ml^−1^ flagella purified from JT1 pWSK_Pba in protein-free blocking buffer (PFBB) (Thermo Fisher, USA), for 2 h. TLC plates were washed three times in Tris-buffered saline (TBS), incubated with anti-Pba flagellin antibodies 1:5,000 in PFBB, washed again and incubated with 1:10,000 anti-rabbit secondary antibodies conjugated to horseradish peroxidase (Sigma-Aldrich, USA) in PFBB for 2 h. Peroxidase activity was detected using Millipore Immobilon Chemiluminescent substrate and visualized with Amersham Hyperfilm ECL (GE Healthcare, USA). As a guide, the TLC plate prior to antibody probing is shown with the lipids marked, as is the antibody-only negative control.

### Plant adhesion assays

Bacterial cultures grown at 28 °C in lysogeny broth with flagella induction (50 µM IPTG) were incubated with freshly excised 4-week-old potato roots, grown in defined phosphate replete media or 0.5 × Murashige and Skoog medium (Merck, USA) where indicated, at a density of OD_600_ of 0.02 (~ 1×10^7^ c.f.u. ml^−1^) in TBS, for 2 h at 20 °C. Loosely adherent bacteria were removed by washing in sterile TBS, and adherent bacteria from macerated tissue were enumerated on solidified LB medium with ampicillin (50 μg ml^−1^ for *E. coli*) or crystal violet pectate medium [19] for Pba1043. Data were collected from three independent experiments with five biological replicates per treatment. Statistical analysis was performed using GraphPad Prism v10.1.2.

## Results

### Functional binding of heterologously expressed Pba1043 flagellin to plant lipids

**Fig. 1.**
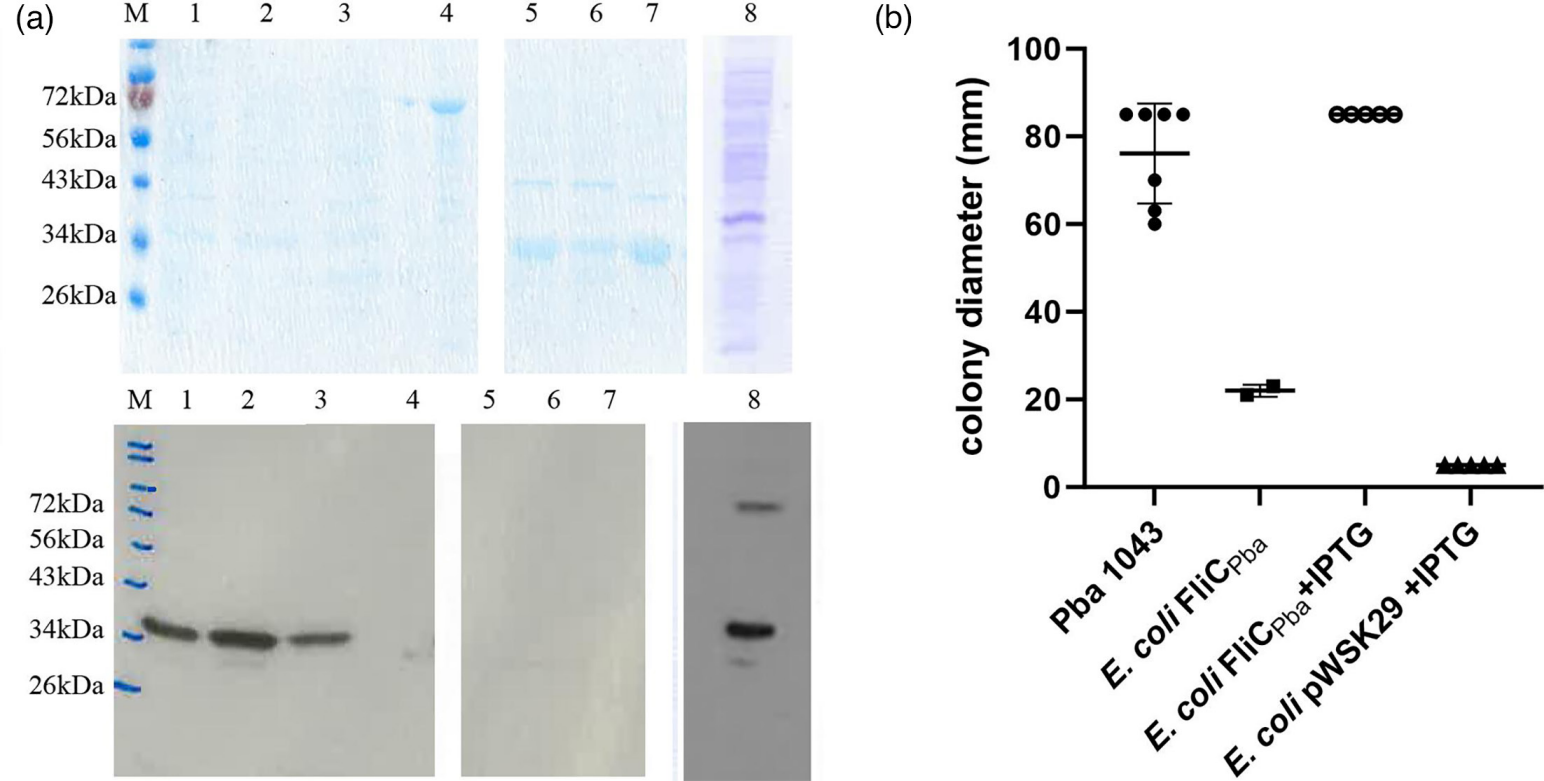
Pba1043 FliC cloning, purification and motility. Antibody specificity against FliC was assessed against purified flagella obtained from a range of Pba isolates, one isolate of *E. coli* O157:H7 and two *Dickeya* spp. (a) A composite gel 12% SDS PAGE Coomassie stained gel (top panel) and corresponding immunoblot (bottom panel) of Pba isolates SCRI_1043 (lane 1), SCRI_1039 (lane 2), SCRI_41 (lane 3), *E. coli* O157:H7 isolate Sakai (lane 4), *Dickeya solani* isolate IPO2222 (lane 5), Pba isolate SCRI_3239 (lane 6), *Dickeya dianthicola* isolate SCRI_3534 (lane 7) and FliC_Pba_ from *E. coli* JT1 (lane 8). (b) Motility of Pba WT isolate SCRI_1043 (Pba 1043), *E. coli* JT-1 expressing pWSK-*fliC*_Pba_ (*E. coli*_Pba FliC) and empty vector control (pWSK29) was assessed from motility agar +/− IPTG induction.

Functional binding of Pba1043 flagella to potato tissue was assessed with the filament flagellin, *fliC*, in isolation from any other factors that may influence binding. We have previously shown that different flagellin proteins (i.e. serotypes) from *E. coli* were all capable of interacting with plant tissue, albeit to varying extents [9]. Therefore, the same approach was used to assess Pba1043. Antibodies were generated from purified Pba1043 flagella and, by western blotting, found to interact with flagella purified from another three Pba isolates, but not the closely related species *Dickeya solani*, *D. dianthicola* or *E. coli* H7 (Fig. 1a), demonstrating specificity. Motility of the transformed *E. coli* strain, JT1+pWSK-*fliC*_Pba_, was found to be comparable to WT Pba (Fig. 1b), but only when expression was induced from the low-copy plasmid with 50 µM IPTG (Sigma-Aldrich, USA). There was no motility for either the uninduced culture or for the strain containing the empty vector (JT1+pWSK29).

**Fig. 2.**
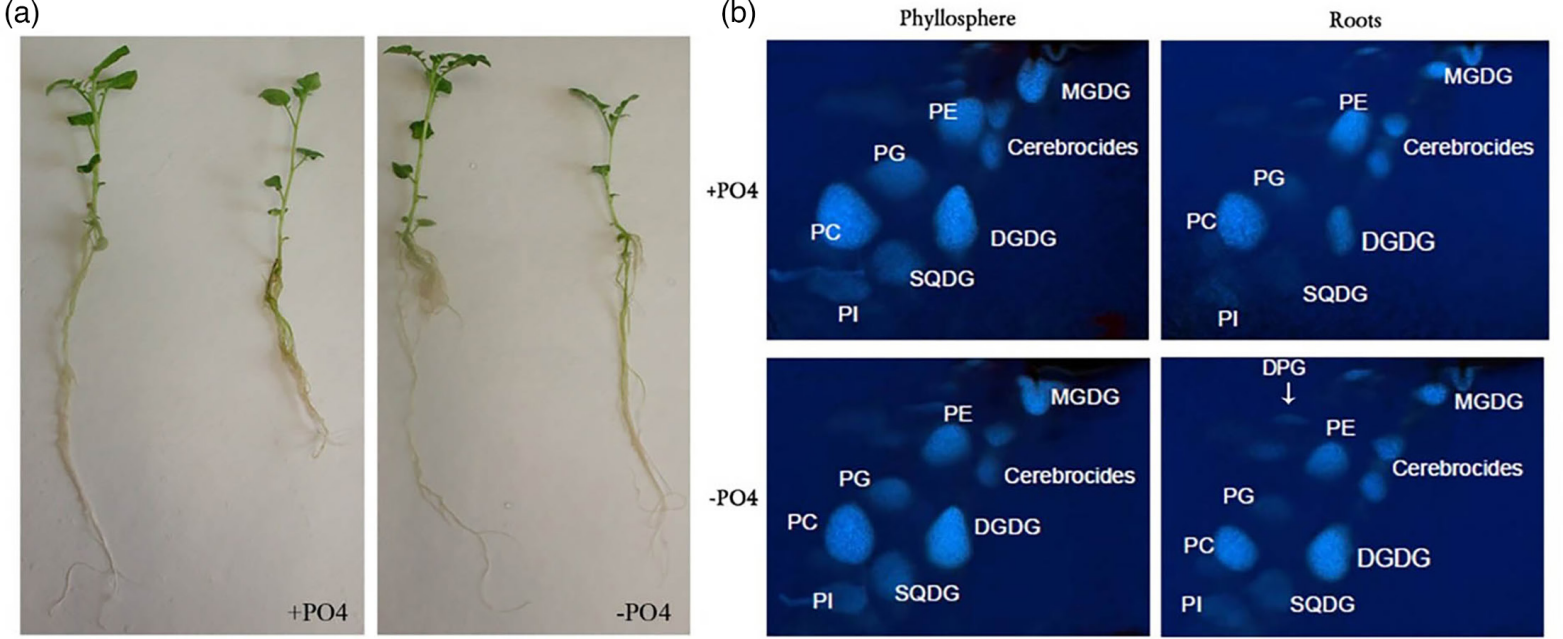
Potato plants (var. Estima) and TLC lipid analysis. Micropropagated plants were grown hydroponically +/− phosphate for 14 days prior to lipid extraction. (a) Representative examples of the largest and smallest plants; (b) potato lipid chromatography, separated by 2D TLC for phosphatidylcholine (PC), phosphatidylinositol (PI), phosphatidylethanolamine (PE), phosphatidylglycerol (PG), diphosphatidylglycerol (DPG), monogalactosyl diacylglycerol (MGDG), digalactosyl diacylglycerol (DGDG) and sulphoquinovosyl diacylglycerol (SQDG).

Once heterologous expression of FliC from *E. coli* JT-1 pWSK_*fliC*_Pba_ was confirmed, functional binding to the roots of potato was tested. Roots have previously been used to characterize Pba biofilm formation on plant tissues [20], and Pba interactions with the rhizosphere of other weed species have been described [21]. Since *E. coli* flagella were previously found to interact with charged plasma membrane lipids and binding was reduced when the proportion of charged lipids was manipulated, the same approach was employed for Pba filament flagellin. Potato microplants were grown in the absence of phosphate to redistribute the relative proportions of charged and uncharged lipids as described previously [9,17] (Fig. 2). 2D TLC showed that potato growth under phosphate limited conditions (-PO_4_) resulted in an increase of charged sulpho- and uncharged galacto-lipids (sulphoquinovosyldiacylglycerol (SQDG), monogalactosyldiacylglycerol and digalactosyldiacylglycerol, respectively) in root and leafy tissues, with a reduction of phospholipids, e.g. phosphatidylcholine (Fig. 2b). Glucuronosyldiacylglycerol was not detectable in this experiment [22], probably due to its very low concentration. As anticipated, growth under phosphate-rich conditions resulted in barely detectable levels of SQDG in the root tissue, since this lipid is normally associated with chloroplasts [17]. However, the presence of some SQDG in the root tissue preparation most likely occurred because of the method of plant cultivation, which resulted in some chlorophyll below the root crown (Fig. 2a).

**Fig. 3.**
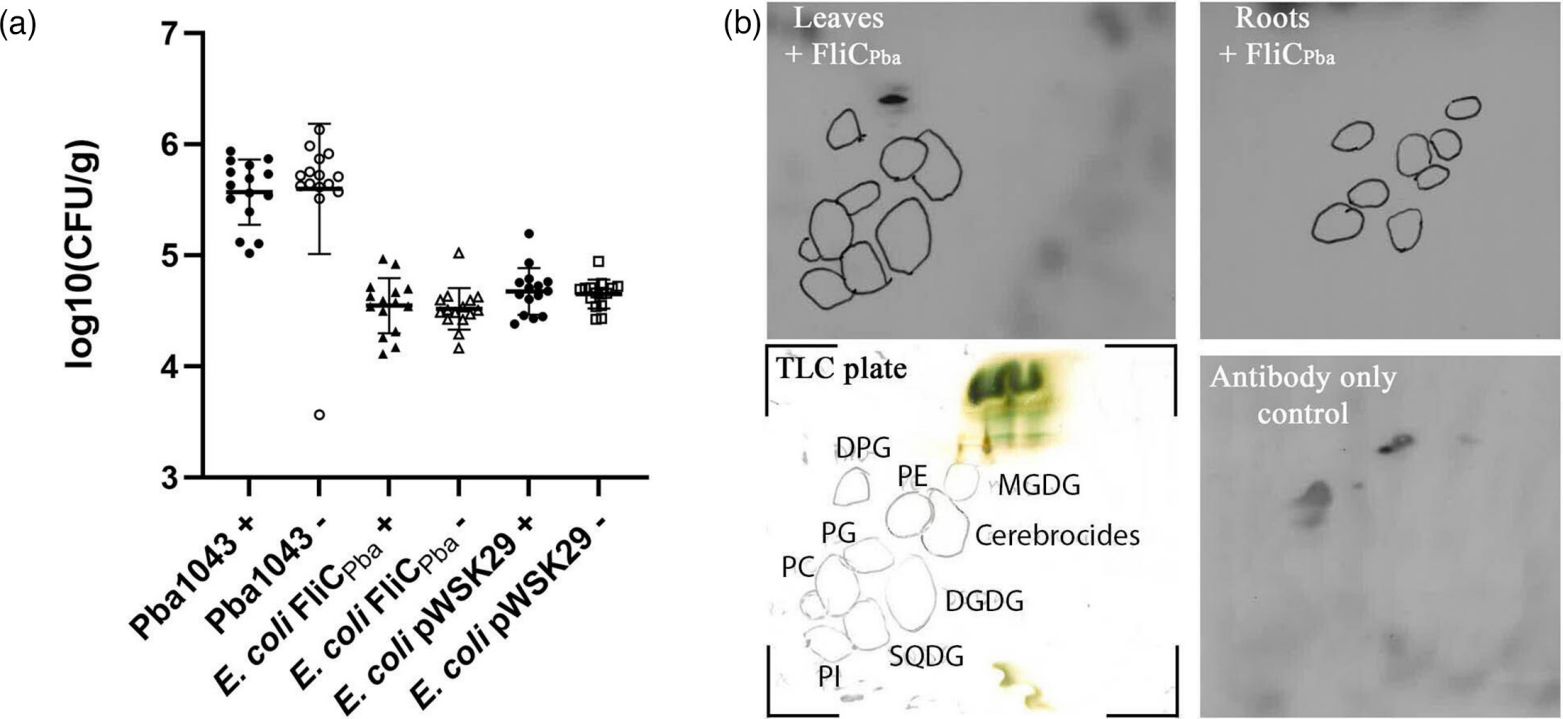
Functional FliC_Pba_ flagella interactions. Functional binding of FliC_Pba_ flagella was assessed (a) on potato roots from plants grown in the presence (+, filled symbols) or absence (−, open symbols) of phosphate and inoculated with Pba1043 or *E. coli* JT1 expressing FliC_Pba_ (FliC) or the vector-only plasmid (pWSK29). (b) Potato lipids extracted from leaves (potato leaves+FliC_Pba_) or roots (potato roots+FliC_Pba_) were resolved by 2D TLC, and FliC_Pba_ interactions tested by far western as described in the ‘Methods’. As a guide, the leaf sample TLC plate prior to flagella probing and immunodetection is shown with the lipid species marked (TLC plate; DPG, diphosphatidyl glycerol; PE, phosphatidylethanolamine; PG, phosphatidylglycerol; PC, phosphatidylcholine; PI, phosphatidylinositol; MGDG, monogalactosyl diacylglycerol; DGDG, digalactosyl diacylglycerol; SQDG, sulphoquinovosyl diacylglycerol), as is the antibody-only negative control.

Once it was established that potato microplant growth under phosphate-limited conditions had altered the lipid composition, flagellin functional binding was assessed from inoculation of *E. coli* JT1 bacteria expressing FliC_Pba_ onto the roots of potato plants. Expression of FliC_Pba_ was found to not affect the numbers of *E. coli* JT1 bacteria recovered from potato roots, with no significant difference between plants grown under phosphate replete (+) or depleted (−) conditions, compared to the empty vector control (Fig. 3a). Although the aim here was to test the Pba filament flagellin protein in isolation, native expression of FliC_Pba_ from WT Pba1043 also did not affect binding to potato roots grown in phosphate-deplete compared to phosphate-rich conditions (Fig. 3a). It was noticeable that the level of Pba1043 recovered from the potato roots was at least one order of magnitude higher than *E. coli*, which is in keeping with our previous data showing that *E. coli* K-12 isolates interact relatively poorly with plant tissue unless endowed with additional colonization factors [9,23]. We confirmed that *E. coli* JT1 expressing *E. coli* H7 flagella also bound to potato roots at least one order of magnitude higher than the control and *E. coli* expressing FliC_Pba_ (Fig. S1, available in the online Supplementary Material), aligning with previous work on other plant species. The absence of functional binding of FliC_Pba_ to potato tissue indicated no physical interaction between the flagellum and the plant tissue. This was assessed biochemically by incubating purified heterologous flagella (i.e. FliC_Pba_ derived from *E. coli* JT1) with potato plasma membrane lipids separated by chromatography and detected with a specific FliC_Pba_ antibody. No direct interaction of the flagella was observed with any of the lipids obtained from potato plants either grown in phosphate-rich (+) or -deplete (−) conditions, supporting the lack of functional binding (Fig. 3b).

### Motility is required for *P. atrosepticum* SCRI1043 attachment to potato tissues

**Fig. 4.**
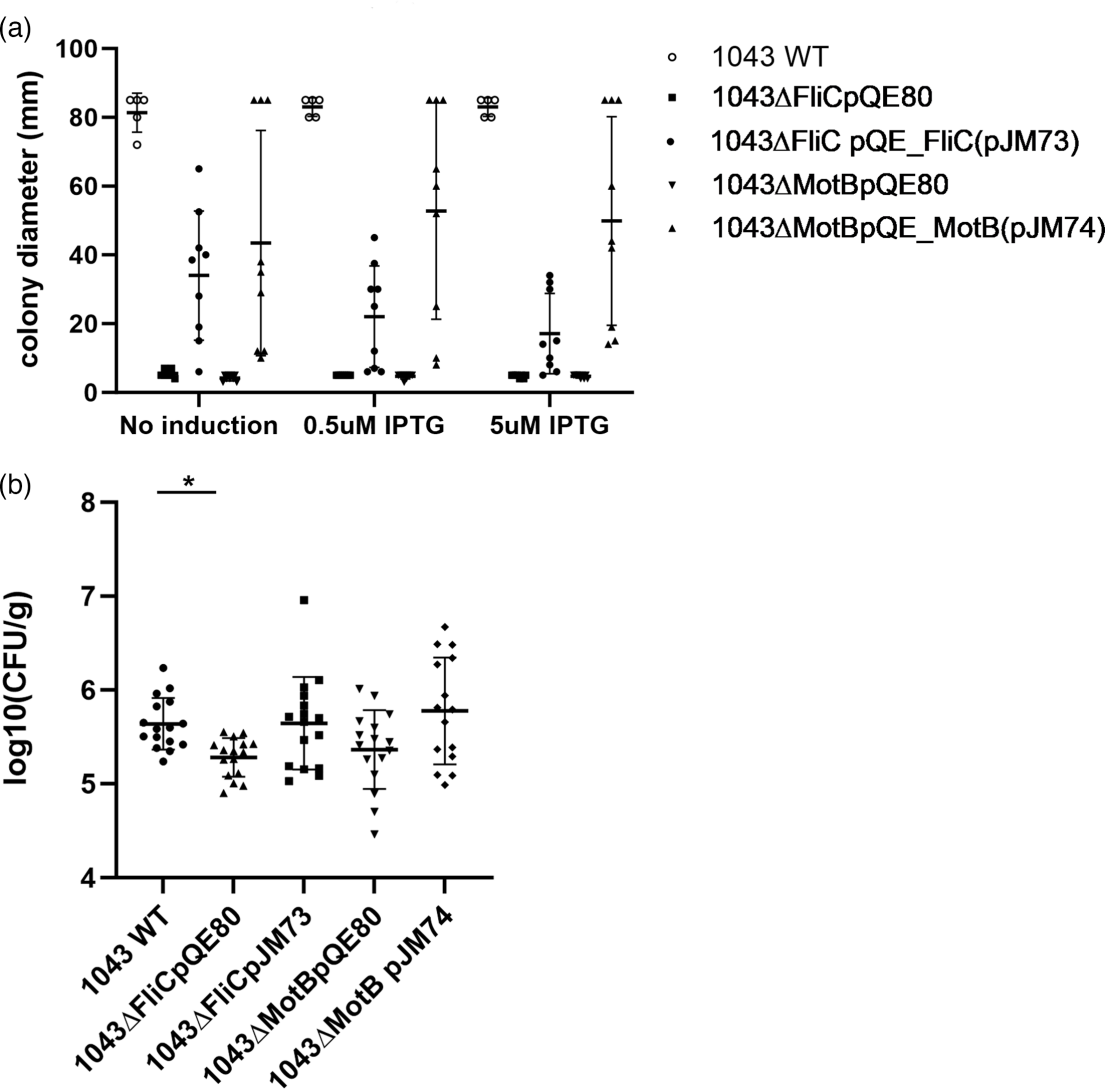
Motility and adherence to potato roots of Pba1043 and motility mutants. Pba1043 deficient in flagellin (*fliC-*) or stator (*motB-*) motility (a) and adherence to potato roots (b) and complemented in trans with plasmids (pJM73=pQE80_*fliC* or pJM74=pQE80_*motAB*) or empty vector (pQE80) control.

To uncouple the role of the flagella for non-specific recognition of host tissue for attachment from motility, we generated a *fliC-* mutant in Pba1043 by homologous recombination. Multiple attempts were made with the same approach to generate a *motA*- or *motAB-* stator mutant, but a successful recombination event could not be recovered. Therefore, we screened a Tn5 library [12], isolated a *motB* mutant and transduced it back into Pba1043 with phiM1. We complemented each mutant in trans, and motility was restored in the Pba1043Δ*fliC* mutant without IPTG induction and in the Pba1043Δ*motB* mutant with 0.5 µM IPTG induction (Fig. 4a). Increasing IPTG concentrations and, thereby, induction negatively impacted motility in the complemented flagellin (*fliC*) mutant in a dose-dependent manner, whereas the *motB* mutant had the largest average colony diameter when induced with 0.5 µM compared to 5 µM IPTG or no IPTG. There was a significant reduction in the mean number of Pba1043 lacking flagella (1043*ΔfliC* pQE80) recovered from potato roots after 2 h incubation compared to WT Pba1043 (*P*=0.02 Kruskal–Wallis test with Dunn’s multiple comparisons test) and attachment was restored to WT levels in trans (1043Δ*fliC* pJM73) (Fig. 4b). For the flagella expressing but motility-deficient mutant (1043Δ*motB* pQE80), there was a slight but not significant (*P*=0.326) reduction in the mean number of bacteria recovered from potato roots compared to Pba1043 WT. When complemented in trans with *motAB* (1043Δ*motB* pJM74), attachment to roots was restored beyond WT levels.

**Fig. 5.**
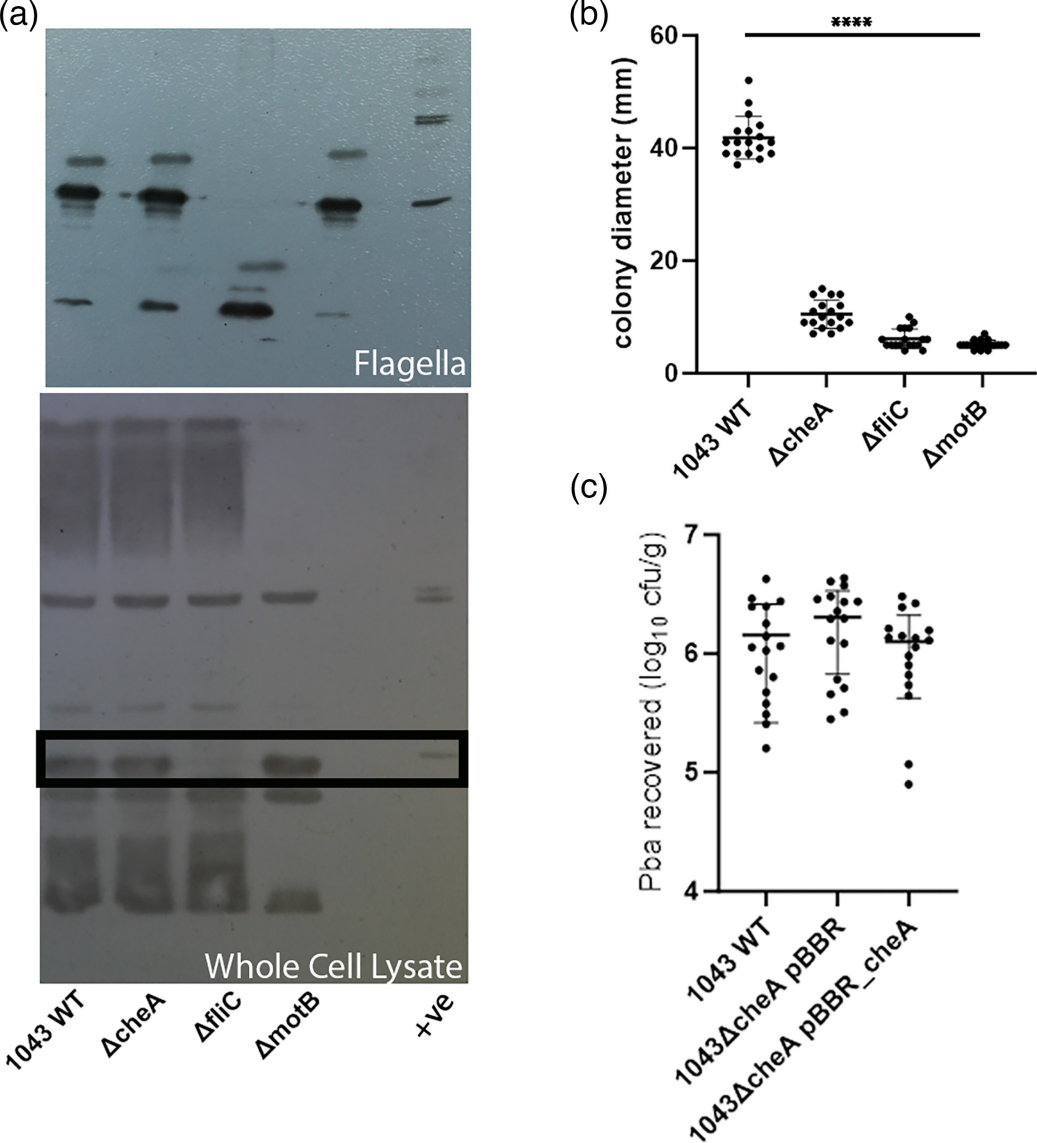
Flagella expression, motility and potato root adherence of Pba1043 chemotaxis mutant. Pba1043 and motility mutants Pba1043Δ*cheA*, Δ*fliC* and Δ*motB* were assessed for flagellin production by immunoblotting bacterial whole cell and flagella preparations (a) and motility on soft agar plates after 48 h incubation (b). Recovery of Pba1043WT, chemotaxis mutant Pba1043Δ*cheA* (empty vector control pBBR) and complemented mutant in trans (pBBR_cheA) from potato roots after 2 h incubation from three independent experiments of at least five biological plant replicates (c).

The chemosensory system has been shown to be involved in the control of bacterial motility [24]. We investigated the effects of a Pba1043 chemosensory defective mutant, lacking the central histidine kinase *cheA*. Western blotting for FliC_Pba_ against flagella preparations and whole cell lysate from Pba1043 and all motility mutants in this study shows that the flagellin is expressed in all isolates except for the defined *fliC* mutant (Fig. 5a). Assessing the Pba1043 *cheA* mutant motility in soft agar assays shows a significant reduction in motility (one-way ANOVA with Tukey’s multiple comparisons test; *P*<0.0001) compared to Pba1043, but it is significantly more motile than Pba1043Δ*fliC* and Pba1043Δ*motB* mutants (Tukey’s multiple comparisons test; *P*<0.0001). There is no significant difference in the mean distance travelled for the Pba1043Δ*fliC* compared to the Pba1043Δ*motB* mutant. Pba1043 is not efficient at forming biofilms on polystyrene under lab conditions [25], and the *cheA* deletion did not improve biofilm formation or expression of multi-repeat adhesin protein (MRP), as assessed by non-pigmentated growth of all isolates on Congo Red (CR) plates [26] (results not shown). To determine if the Pba1043Δ*cheA* motility impairment, compared to Pba1043 WT, affected the bacteria’s capacity to adhere to potato roots, we compared a vector complemented mutant (pBBR_*cheA*) to an empty vector control (pBBR) and Pba1043WT. Fig. 5c shows that the mean number of Pba1043Δ*cheA* (pBBR) recovered from potato roots was not significantly (*P*=0.269, Kruskal–Wallis test) different from that of Pba1043 WT or the complemented Pba1043Δ*cheA* (pBBR_*cheA*)

## Discussion

Pba and *E. coli* have been found to share several aspects of plant colonization [27], which is perhaps unsurprising given their genetic relatedness [28]. A major difference is in their ability to cause disease on plants, in that while *E. coli* can colonize plants, Pba is a disease-causing pathogen on potato [21]. In our study, we found that heterologous expression of Pba flagellin protein FliC did not provide an advantage for bacterial adhesion to potato tissues or specifically recognize ionic lipids, yet Pba1043 adhered an order of magnitude greater than *E. coli* JT1 (Fig. 3a). Growing potato microplants in phosphate-deplete media did not affect the mean number of Pba1043 recovered from potato roots, showing that Pba1043 adhesion via native flagella, or other adhesins, was not altered with an increase in ionic lipid composition in plant plasma membranes (Fig. 3a). Pba1043 flagella differ from *E. coli* as they are shorter in amino acid sequence, lacking the hypervariable regions in domains 2 and 3, and may have post-translational modifications via a seven gene flagellin glycosylation island between *fliA* and *fliC* in its genome [9,12, 29]. Glycosylation of Pba flagella does not have an impact on motility, as demonstrated from the heterologous expression of FliC_Pba_ by *E. coli* JT1, restoring motility to Pba1043 levels (Fig. 1). Flagellin glycosylation has previously been shown in related species *Dickeya dadantii* 92–31 and *Pectobacterium carotovorum* EC1 [30], but any potential role in *Pectobacteriaceae* pathogenicity remains to be characterized. We aim to investigate this in future studies.

As adherence can be a function of multiple surface-associated factors, defined motility mutants in Pba were characterized to uncouple potential physical interactions (Δ*fliC*) from motility function (Δ*motB*). A flagella-deficient Pba1043 (Δ*fliC*) was significantly reduced in its adhesion to potato roots, and an immotile, but flagellated Pba1043 (Δ*motB*) was reduced to comparable, albeit not significant, levels. Complementation of the motility mutants in trans showed an additive effect for *motAB* complementation (Fig. 4b). This demonstrates that flagella contribute to Pba1043 adherence to plant tissues indirectly through their motility function, which has also been reported for fish pathogen *Aeromonas hydrophila* [31]. Flagella are involved in host adhesion in numerous host–pathogen interactions, but few studies have uncoupled flagellar motility from direct interactions. A study by Gorski *et al*. investigated *Listeria monocytogenes* adherence to plant sprouts and reported that adhesion was impacted only in a flagellin (*flaA*) mutant, not an immotile flagellated *motAB* mutant, indicating a direct flagella–plant interaction as the shaking conditions in their adherence assay complemented the lack of motility from the *motAB* mutant [8]. Swimming motility was shown to be required for Pba pathogenicity through a mutant screen [32] and deletion of prophages [33].

Motility is also essential for chemotaxis, and it has been previously characterised that chemoreceptor mutants in *Pectobacterium brasiliense* Pb1692 are reduced in attachment to potato leaves [34]. In this study, we show that a chemotaxis mutant, Pba1043Δ*cheA*, can produce and assemble flagella (Fig. 5a), is motility-impaired, not immotile (Fig. 5b), and can adhere to potato roots to comparable levels as Pba1043. CheA is the sensor kinase that is phosphorylated by the chemoreceptors in the presence of attractants. CheA transfers its active phosphate to response regulator CheY, which modulates flagellar motor activity among other factors, including type IV pili-mediated motility, biofilm formation or other alternative cellular functions depending upon the bacterial strain (reviewed in [35]). Biofilm formation on polystyrene was not affected by the mutation of *cheA,* nor did it produce a CR^+^ phenotype, which would indicate an increase in c-di-GMP levels or expression of other surface-associated α-d-glucopyranosyl units, basic or neutral polysaccharides, or proteins such as MRP [25,26]. Attachment to host tissues is a multifactorial process and the MRP has previously been characterized for Pba adherence to potato root tissues [26].

The regulatory cross-talk between flagella-mediated motility, chemotaxis and expression of surface adhesins in Pba1043 warrants further investigation. This work raises questions about differences in the molecular interactions between bacteria and host plants, with implications for the outcome that are relevant to both crop protection as well as food safety.

## Supplementary material

10.1099/mic.0.001588 Uncited Fig. S1.

## Abbreviations

CR: Congo Red
MRP: multi-repeat adhesin
PFBB: protein-free blocking buffer
SQDG: sulphoquinovosyldiacylglycerol
TBS: Tris-buffered saline
TLC: thin layer chromotography
