# Supplementary material for: Pectobacterium atrosepticum SCRI1043 flagella mediate adherence to potato plants indirectly through motility
Source: Microbiology (Reading). 2025 Jul 30;171(7):001588. doi: 10.1099/mic.0.001588 (PMC12310338; doi:10.1099/mic.0.001588)
Supplement: Uncited Fig. S1. [file mic-171-01588-s001.pdf]

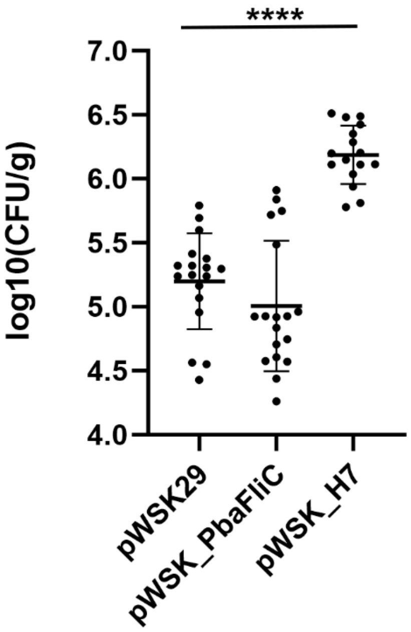

Figure S1: Functional binding of flagella to Estima potato roots.

Potato microprops were grown in 0.5x MS media for 14 days before the roots were incubated with *E. coli* JT1 expressing $FliC_{Pba}$ (pWSK_PbaFliC), $FliC_{H7}$ (pWSK_H7) or empty vector control (pWSK29) for 2 hours. *E. coli* expressing $FliC_{H7}$ (pWSK_H7) are significantly more adherent to potato roots than when expressing $FliC_{Pba}$ (pWSK_PbaFliC) or the vector-only control ($p < 0.0001$, Kruskal-Wallis test).
